# Comparison of pain catastrophizing and anxiety in patients with dyspareunia and healthy women: a cross-sectional study

**DOI:** 10.25122/jml-2022-0234

**Published:** 2023-02

**Authors:** Zahra Mohammadzadeh, Zohreh Khakbazan, Maryam Rad, Farnaz Farnam

**Affiliations:** 1 Reproductive Health and Midwifery Department, Tehran University of Medical Sciences, Tehran, Iran; 2 Social Security Organization, East Azerbaijan Treatment Management, Tabriz, Iran; 3 Oral and Dental Diseases Research Center, Kerman University of Medical Sciences, Kerman, Iran

**Keywords:** sexual pain, Genito-Pelvic Pain/Penetration Disorder, dyspareunia, pain catastrophizing, anxiety, Iran, D – Dyspareunia, GPPPD – Genito-Pelvic Pain/Penetration Disorder, PC – Pain Catastrophizing Scale, PF – Pain-Free, STAI – Spielberger State-Trait Anxiety Inventory-6, WHO – World Health Organization

## Abstract

Despite the prominent role of cognitive-affective variables, such as pain catastrophizing and anxiety in chronic pain, little is known about their relationship with dyspareunia. This study compared pain-catastrophizing and anxiety in reproductive-aged women with and without dyspareunia. A controlled cross-sectional study was conducted on 398 married women in Iran selected by convenience sampling. Sampling was performed both online and in person. Data were collected using a checklist designed for the study, including background characteristics, self-reported dyspareunia, and two standard questionnaires: the Pain Catastrophizing Scale (PCS) and the Spielberger State-Trait Anxiety Inventory-6 (STAI-6). Results showed that 49.5% of the women reported dyspareunia in the previous six months, with a reduced figure of 42% and 31% when using more specific criteria for dyspareunia. Women with dyspareunia had significantly higher scores for pain catastrophizing and anxiety than the control group without dyspareunia. Pain-catastrophizing was associated with aversion to genital contact and body image dissatisfaction. Anxiety was correlated with age, marriage duration, and sexual abuse. Managing anxiety and catastrophizing thoughts may help dyspareunia patients better cope with pain.

## INTRODUCTION

Dyspareunia is a prevalent sexual dysfunction among women, characterized by recurrent or persistent genital pain that occurs before, during, or after vaginal intercourse. This disorder is often underdiagnosed and undertreated and can significantly impact a woman's quality of life [1, 2]. A systematic review by the World Health Organization (WHO) reported the global prevalence of dyspareunia ranging from 8% to 22% [3]. An estimated one-fifth of women develop this disorder at some point in life [4]. In one study conducted on women from 29 different countries, the prevalence of dyspareunia was much higher in Asia than in other continents and was reported as 21% in the Middle East [5]. There are different statistics on the prevalence of dyspareunia in Iran. In a review study by Nasehi *et al*. (2017), the prevalence of dyspareunia varied widely from 9% to 95.9% [6]. In a population-based study in Iran, Farnam *et al*. (2018) reported the prevalence of severe dyspareunia based on the Genito-Pelvic Pain/Penetration Disorder (GPPPD) criteria as 10.5% and mild dyspareunia as 26%. In their study, 33% of the women reported having experienced dyspareunia over the previous six months [7].

Dyspareunia is a multifactorial problem involving various physiological and cognitive-affective components [8]. The etiology of dyspareunia can be attributed to cognitive-affective factors (such as sexual abuse, fear, anxiety, and pain catastrophizing), biomedical factors (such as hormonal changes, infections, sexually transmitted diseases, and pelvic floor muscle disorders), cultural-religious factors (such as attitude toward sexuality), and relational factors (such as the sexual partner’s reactions) [9]. Identifying the factors contributing to dyspareunia is essential for successfully diagnosing and treating this disorder [10].

Dyspareunia significantly impacts women's physical and mental health and is associated with several individual effects such as embarrassment, shame, guilt, lack of self-esteem, sense of deprivation, anxiety, anger, and depression [11, 12]. Dyspareunia also causes interpersonal problems such as decreased sexual satisfaction and function and significant distress and conflict between couples [12, 13]. Despite the critical role of cognitive-affective variables such as pain catastrophizing (PC) and anxiety in the etiology of dyspareunia [4], there are contradictory results on the association between these factors and dyspareunia [14].

PC is a psychological trait referring to the tendency to ruminate, magnify, or feel helpless about pain, and it is observed in people who show a negative and exaggerated reaction to a painful experience. PC is also considered a help-seeking behavior characterized by magnifying illness in a social context that does not necessarily aim to reduce pain [15, 16]. People with higher PC scores tend to magnify the pain, cannot divert their attention from it and are incapable of managing pain [16]. The relationship between PC and chronic pains such as vaginismus is clear; still, there is little information available about the relationship between PC and dyspareunia, and literature on the subject is contradictory [17, 18].

Anxiety is described as a feeling of panic and worry that is transient in nature [19] and has several physical and psychological symptoms. All people might show some degree of anxiety in the face of stress or danger, but some people experience severe anxiety in their daily activities that can cause distress and significant disruption in normal life [20]. Some studies suggest that anxiety is not only considered an independent predictor of dyspareunia but can also be one of its consequences [4, 14].

Since dyspareunia has detrimental effects on women's health, it is imperative to research its contributing factors and appropriate treatments. Although the number of studies examining dyspareunia has grown over the past decade, their findings on the factors associated with dyspareunia are contradictory, and there is a need for further research. Furthermore, most studies on this subject have been conducted in Western countries, and few standard studies have been carried out on dyspareunia in traditional and collectivist countries [18]. The critical role of psychological factors such as PC and anxiety in the etiology of dyspareunia [4] and the modifiable nature of these factors demonstrate the need for further research on this subject [14].

This paper is part of a larger research project on dyspareunia in women of reproductive age. The effects of dyspareunia on sexual health have been reported in another paper [13]. The current study aimed to compare PC and anxiety in women with and without dyspareunia and determine the prevalence of dyspareunia among the study sample.

## MATERIAL AND METHODS

This cross-sectional controlled study was conducted from Dec. 2020 to May 2021 in Iran and complied with the principles of the Declaration of Helsinki.

### Recruitment

The study utilized a convenience sampling method, recruiting married premenopausal women as participants. To minimize selection bias and ensure a diverse sample, data collection was done through both online and in-person methods. By including both online and in-person recruiting, the sample covered a broader range of women, including those who may not have access to or proficiency in using smart devices.

The online questionnaire was designed on a web-based secure server (Porsline). This server kept the identities of the participants confidential and allowed them to send the questionnaires back to the researchers anonymously. To achieve maximum heterogeneity, online questionnaires were distributed across different groups of women in the country via Telegram, WhatsApp, and Instagram. Due to the ongoing COVID-19 pandemic at the time of the study, face-to-face sampling was performed only in one city, and women visiting private or public health centers in that city were recruited. After thoroughly explaining the objectives and confidentiality measures of the study to potential participants, the inclusion criteria were rigorously assessed for each candidate. Eligible women willing to participate in the research completed an informed consent form. The average time to complete the questionnaire was 10 to 15 minutes. After completing and sending the questionnaires to the research team, the women who answered ‘yes’ to both questions asking about “the existence of dyspareunia over the last six months” and “pain in ≥50% of intercourses” were taken as cases of dyspareunia (D), and the other women were taken as pain-free (PF) and were therefore recruited in the control group.

The eligibility criteria for both groups included being premenopausal, married, non-pregnant, having had intercourse with the husband at least 5-6 times over the previous six months, and not having given birth during the preceding six weeks.

### Outcome measurements

The data collection instrument was a checklist designed for this study, including background characteristics, two items to assess dyspareunia, and two standard questionnaires on PC and anxiety.

The checklist of background characteristics included 12 items on variables such as age, duration of marriage, years of education (counting from the first grade of elementary school), occupation, economic status, history of diseases, medication use, sexual abuse, body image satisfaction, aversion to genital contact, and the duration and location of the pain. These factors were each assessed with a single item on a self-report basis.

To diagnose dyspareunia, the participants answered ‘yes’ or ‘no’ to two items: “the existence of dyspareunia over the last six months” and “pain in ≥50% of intercourses”. If they answered ‘yes’ to both questions, they would be recruited to the dyspareunia group.

Pain Catastrophizing: The 13-item Pain Catastrophizing Scale (PCS) was administered to assess the respondents’ catastrophic thoughts and behaviors toward pain. The responses were given a 5-point Likert scale, with a score of 0 for "not at all" and 4 for "all the time". The overall score ranged from 0 to 52, with higher scores indicating a higher level of catastrophic thoughts and feelings. The reliability and validity of the instrument have been confirmed in previous studies with Cronbach’s alpha of 0.87 [21].

Anxiety: Spielberger State-Trait Anxiety Inventory-6 (STAI-6) was administered to measure anxiety [22]. This questionnaire consists of six items on a 4-point Likert scale. The "almost never" option received a score of 1, and the "almost always" option a score of 4, showing high anxiety. Items 1, 4, and 5 are reverse-scored, and the overall score is between 6 and 24. This short six-item version has the same full factorial structure and reliability as the original 20-item questionnaire (r=0.95). The validity and reliability of this questionnaire were confirmed with Cronbach's alpha of 0.82 [23].

### Statistical analysis

Since the initial study was very extensive and measured many variables, and based on experts’ recommendations, a sample of 15-20 was allocated to each variable [24]. The final sample size was estimated as 400, 10% of which would be reached through in-person sampling. After sending out the questionnaires to the target groups and based on the two questions used for diagnosing dyspareunia, the participants were divided into two groups: a group with and a group without pain. Based on the protocol, the two groups were matched in age (34 vs. 34.4 years). Data analysis was performed in SPSS 22 (IBM, Armonk, NY, USA). The frequency distribution and mean values (standard deviation) were obtained using descriptive statistics, and the background characteristics related to dyspareunia were analyzed using inferential statistics (independent t-test, Chi-square test, and Fisher's exact test). A multiple logistic regression analysis was also performed using the Enter method to estimate the strength of the association of PC and anxiety with the background characteristics.

## RESULTS

A total of 398 women entered the final analysis, including 167 in the dyspareunia (D) group (144 online and 23 in-person samples) and 231 in the pain-free (PF) group (214 online and 17 in-person samples).

Moreover, 197 women (49.5%) had experienced dyspareunia based on their response to the item “the existence of dyspareunia over the last six months”. By also including the item of “pain in ≥50% of intercourses”, the frequency of dyspareunia was reduced to 42% (n=167). Since this study aimed to assess people with dyspareunia, the latter 167 samples made up the final case (dyspareunia) group. Taking “sexual distress” as a third criterion for diagnosing dyspareunia further decreased the frequency of this disorder to 31%. Table 1 presents the background characteristics of the two groups.

**Table 1:** Distribution of contextual factors in dyspareunia and pain-free women.

Characteristic	Dyspareunia (D) (N=167)	Pain-free (PF) (N=231)	P-value
	M (SD)	M (SD)	
**Duration of marriage (yr)**	11.9 (7.0)	11.8 (6.6)	0.93 ^ƚ^
**Duration of the pain (yr)**	5.3 (6.4)		
**Education years from primary school**	13.5 (3.0)	14.4 (3.1)	0.006 ** ^ǂ^
	**N (%)**	**N (%)**	
**Age (yr)**			
30>	42 (25.1)	56 (24.2)	0.760 ^ǂ^
40-30	98 (58.7)	131 (56.7)	
40<	27 (16.2)	44 (19.0)	
**Women occupation**			
Household	116 (69.5)	149 (64.5)	0.555 ^ǂ^
Part-time job	27 (16.2)	41 (17.7)	
Full-time job	24 (14.4)	41 (17.7)	
**Financial situation**			
Very poor	6 (3.6)	7 (3.0)	0.430 ^ǂ^
Poor	14 (8.4)	16 (6.9)	
Within middle range	87 (52.1)	114 (49.4)	
Good	54 (32.3)	75 (32.5)	
Very good	6 (3.6)	19 (8.2)	
**Location of the pain**			
Vaginal entrance	59 (41.5)	-	-
Vaginal depth	41 (28.9)	-	
Both	42 (29.6)	-	
**History of any disease**			
None	59 (35.5)	129 (55.8)	p<0.001*** ^ǂ^
Gynecological diseases ^a^	68 (41.0)	58 (25.1)	
Anxiety and depression	39 (23.5)	44 (19.0)	
Cancer	1 (0.6)	0 (0.0)	
**Taking medication**			
No	120 (71.9)	196 (84.8)	0.002** ^ǂ^
Yes	47 (28.1)	35 (15.2)	
**History of sexual abuse**			
No	161 (96.4)	220 (95.2)	0.569 ^ǂ^
Yes	6 (3.6)	11 (4.8)	
**Satisfaction with body image**			
Very Dissatisfied	13 (7.8)	10 (4.3)	0.011* ^ǂ^
Somewhat dissatisfied	14 (8.4)	14 (6.1)	
Moderately	48 (28.7)	75 (32.5)	
Somewhat satisfied	60 (35.9)	58 (25.1)	
Very satisfied	32 (19.2)	74 (32.0)	
**Aversion to touching the genitalia**			
No	139 (83.2)	204 (88.3)	0.147 ^ǂ^
Yes	28 (16.8)	27 (11.7)	

M: Mean, SD: Standard Deviation, ^ƚ^ Analyzed by the Independent t-test, ^ǂ^ Analyzed by the Pearson chi-square, P<0.05*, p<0.01 **, P<0.001***, a Endometriosis, Fibroma, Vaginal infectious diseases.

The education level of group D was significantly lower (P=0.006) than the PF group. Most participants were in the 30-40 years age group. The majority of women in both groups were housewives, and the economic status of about half of the women was average. History of diseases, medication use, and body image differed significantly between women with and without dyspareunia. The most common disease reported in both groups was gynecologic disease. In the D group, the average duration of pain was 5.3 years, and the majority of the women in this group (41.5%) reported pain at the vaginal opening.

Regarding the two main variables of the study, the results presented in Table 2 show that the mean scores of PC and anxiety were significantly higher in the D group (P<0.001) than in the PF group. In the D group, the mean score of PC was about 8 points (15.5%) higher than in the PF group, and their mean anxiety score was about 2 points higher.

**Table 2:** Comparison of cognitive-affective variables in dyspareunia and pain-free women.

Characteristic	Dyspareunia (N=167) M (SD)	Pain-free (N=231) M (SD)	P-value
**Pain catastrophizing**	22.4 (13.2)	14.6 (11.9)	0.001*** ^ƚ^
**Anxiety**	8.7 (4.9)	7.0 (4.5)	0.001** ^ƚ^

M: Mean, SD: Standard Deviation, ^ƚ^ Analyzed by the Independent t-test, P<0.05*, p<0.01 **, P<0.001***.

A multivariate regression analysis was performed by the Enter method to reveal the factors associated with PC and anxiety in people with dyspareunia (Table 3). PC had a significant relationship with aversion to genital contact and was also linked to body image satisfaction (P=0.09). Aversion to genital contact and body dissatisfaction increased PC by 15.8 and 4.94 points, respectively.

**Table 3:** Correlation of contextual factors with PC and anxiety in women suffering from dyspareunia (n=167).

	B	S.E.	P-value	Beta
**Pain Catastrophizing**				
Aversion of genitalia touching	15.80	7.65	0.051	0.45
Body image satisfaction	4.94	2.78	0.090	0.46
**Anxiety**				
Age	-0.59	0.17	0.003	-0.84
Marriage duration	0.34	0.12	0.014	0.52
Sexual abuse	5.61	3.11	0.086	0.24

B: Unstandardized Beta, SE: Standard Error.

In women with dyspareunia, anxiety is significantly or almost significantly associated with age, duration of marriage, and history of sexual abuse. Each year of increase in age reduced the anxiety score in the D group by 0.59. Also, with each additional year of marriage, the anxiety score increased by 0.34. A history of sexual abuse increased the anxiety score by 5.61.

## DISCUSSION

This study revealed that women with dyspareunia further catastrophize pain and have higher anxiety than pain-free women. The frequency of dyspareunia was reported at 42% in the present study, which is in line with the figures reported by studies conducted in other traditional and religious countries in the Middle East. The prevalence of dyspareunia was reported at 40.5% in Egypt [25], 42.9% in Turkey [26], and 33% in Iran [7]. In a cohort study conducted in the Netherlands over six years, the incidence of dyspareunia was higher than other disorders and was estimated at 28.5% [27]. Studies by Nobre in 2015 [28] and Mitchell in 2017 [29] reported a much lower prevalence of dyspareunia (9.8% and 7.5%, respectively). This difference in prevalence is not only attributed to the different criteria chosen for diagnosing dyspareunia but also to cultural-religious differences and their role in the formation of dyspareunia, which justifies the high prevalence of dyspareunia in Middle Eastern countries and Islamic societies such as Iran [30, 31].

In the present study, the mean PC score of women with dyspareunia (D) was about 8 points (15.5%) higher than pain-free (PF) women, which constitutes a significant difference. PC is a negative affective response regarded as an unfavorable psychological predictor of pain [16, 32]. Despite the lack of comprehensive knowledge about the exact mechanism by which pain catastrophizing is associated with pain, it is evident that this behavior can cause changes in the pain control pathways by increasing the activity of the pain processing regions of the brain and enhancing central sensitization [33]. PC apparently intensifies the experience of pain by creating bias and extreme attention to pain [34]. One justification for this finding might be the significantly lower education in the D group compared to the PF group; higher education is associated with a better understanding of life circumstances and access to information and facilities and higher self-confidence [35], thereby diminishing the catastrophic interpretation of pain. Other studies have also demonstrated the mutual relationship between education and PC [36].

Another explanation for the higher PC in the present study might be the higher incidence of various diseases in the D group (64.7%) compared to the PF group (44.2%). In other words, more exposure to diseases might be accompanied by a more catastrophic reaction to pain. Similarly, PC was more common in people with chronic pain in several studies [37, 38]. Nevertheless, some other studies found no association between PC and dyspareunia [17, 32]. This disparity can be attributed to people's different perceptions of pain.

In the present study, anxiety was significantly higher in women with dyspareunia. Anxiety is, in fact, the most common form of psychological distress in women with dyspareunia [39]. Some socio-cultural factors can further intensify the anxiety induced by dyspareunia. For instance, in traditional and collectivistic societies where men's sexual desires are more important than women's, women with dyspareunia experience more shame and become burdened with a sense of disability [18]. This inner sense of disability and the sexual problems experienced by these women aggravate their anxiety [40].

It should be noted, however, that it is not possible to postulate a cause-and-effect relationship from the present study, and anxiety may be either a risk factor or a consequence of dyspareunia [41]. In other words, anxiety induced by sexual intercourse may cause dyspareunia. In most traditional societies, the first sexual intercourse takes place after marriage, and due to the lack of training and the misconceptions about sex, the first sexual encounter between a married couple is associated with considerable anxiety and fear. Anxiety leads to stiffness of the body and primary dyspareunia, which persist in many cases [42]. In the current study, the significantly higher incidence of various diseases, medication use, and body image dissatisfaction in the D group compared to the PF group can explain the greater anxiety in the D group. According to Melis, there is a direct link between diseases and body image dissatisfaction [43].

Several studies on women with dyspareunia also reported higher anxiety levels in this group of patients [7, 44, 45]. In contrast, other studies reported no association between dyspareunia and anxiety [14, 46]. These discrepancies might be partly attributed to the etiology of dyspareunia, as different individuals have different degrees and experiences of anxiety [47].

This study has several limitations, including the reliance on self-reported data obtained from questionnaires to assess dyspareunia and associated factors, as well as the limited number of items used to diagnose the disorder. Additionally, the COVID-19 pandemic posed challenges in terms of obtaining in-person samples from across the entire country. The cross-sectional nature of the study is another limitation that does not allow for a causal relationship to be drawn between the variables. In other words, this study does not determine whether pain catastrophizing and anxiety are antecedents or consequences of dyspareunia. The strengths of this study include having a control group, an acceptable sample size, and preventing sampling bias by incorporating in-person sampling in addition to online sampling and thus also examining women who did not have access to online questionnaires.

## CONCLUSION

The results of this study revealed the high frequency of dyspareunia among women and the key role of cognitive-affective variables such as pain catastrophizing and anxiety in dyspareunia. In traditional and patriarchal societies such as Iran, meeting the sexual needs of men is much more important than meeting those of women. The values and culture governing such societies can cause a lack of sexual self-confidence in women, which is accompanied by significant anxiety. The impact of dyspareunia on women's health and inter-marital relationships has become more dire in such societies. The significant association of dyspareunia with pain catastrophizing and anxiety in the present study indicates that although dyspareunia is often attributed to physical causes, psychological factors such as pain catastrophizing and anxiety, which are rooted in fear, seem to highly affect this disorder too and should not be neglected. Therefore, diagnostic and therapeutic approaches should include the potential physical and psychological factors affecting the development of this disorder so that all the relevant resources available can be exploited for an optimal treatment outcome. In many cases, it is not possible to entirely eliminate the pain, but if anxiety or pain catastrophizing are present, the patient may be able to better adapt to her pain by managing these factors.

## Data Availability

Further data is available from the corresponding author on reasonable request.

## Personal thanks

The research team is very grateful to all women who played a key role in this study.
